# Hangry bees: Pollen dearth impacts honey bee (*Apis mellifera*) behavior and physiology

**DOI:** 10.1371/journal.pone.0338712

**Published:** 2026-01-16

**Authors:** Elizabeth M. Walsh, Arián Avalos, Kate Ihle, Pierre Lau, Michael Simone-Finstrom, Anabelle A. Acosta, Mandy Frake, Sharon O’Brien, Giovanni Tundo

**Affiliations:** 1 United States of America Department of Agriculture-Agricultural Research Service, Honey Bee Breeding, Genetics, and Physiology Research Unit, Baton Rouge, Louisiana, United States of America; 2 Pollinator Health in Southern Crop Ecosystem Research Unit, United States of America Department of Agriculture-Agricultural Research Service, Stoneville, Mississippi, United States of America; University of Alberta, CANADA

## Abstract

Nutritional deprivation is known to contribute to increased honey bee mortality, physiological stress, aberrant behaviors, and disease incidence. To investigate the effect of a realistic nutritional protein deficiency, we simulated a pollen dearth in half of our experimental colonies by robbing incoming foragers of their pollen loads, the primary source of dietary protein, at the colony entrance. We then conducted temperament assays on each colony weekly for pollen deprived and control counterparts. We also identified the plant species bees foraged from and took various physiological measures of honey bee nutritional status including gland size, lipid quantification, and gene expression to further investigate and explain our behavioral results. We found that colonies deprived of pollen reacted by becoming more defensive and that immature bees likely receive cues during rearing which prime their gene expression and behavior as adults, ultimately suggesting that environmental stress caused significant behavioral changes. Temperament is primarily associated with genotype in the literature, but there are environmental cues that are less acknowledged and still important as our study shows. As droughts become increasingly frequent and resource availability therefore changes over time, the impacts on behaviors of agricultural keystone species need additional consideration in order to form scientifically driven best management practices.

## Introduction

One vital livestock service is pollination, which is largely accomplished by commercially kept honey bees (*Apis mellifera*, L) [1–3]. The docile nature of colonies and ability of beekeepers to breed for gentleness, paired with current large scale transportation and deployment at minimal cost, has allowed a long history of human management [1].The ease of honey bee colony management is a critical factor for their role as the mobile, generalist pollinators which modern agriculture currently requires for food security [1,2]. Colony defense is an emergent trait relying on concerted communication and response to potential threats by individuals in a colony [4]. Defensive colonies are those which exhibit a suite of behaviors, which include stinging and pursuit of potential threats [5]. The variation in intensity of colony defense has been studied both within [6,7] and across populations over the past few decades [8–10], and its genetic underpinnings have recently begun being explored [5,11–14].

There is some information linking honey bee defensive behaviors resulting from environmental factors such as disease state, colony manipulation, alarm pheromone presence, and robbing [10,15–17]. However, these have not been studied as extensively as the impact of genetic components and background on temperament [9–15,18,19]. Historical work has largely focused on the relationships between genetic components and defensive behaviors, perhaps in part due to both the obvious nature of defensive traits as well as the importance of managing these behaviors for safety through breeding practices [9–15,18,19]. This relative lack of knowledge regarding environmental factors which drive temperament is problematic in light of select regulatory practices which solely utilize genotype to exclude particular genotypes from certain regions of North America. These regulations exert additional pressure on honey bee breeders to exclude specific genotypes without adequate consideration of phenotypic temperament. In the context of our rapidly changing environment, it is important to discover how environmental variables impact colony behaviors like temperament in order to form science-based regulatory and breeding practices.

One critical environmental factor for honey bee colonies is nutritional availability, which highly varies across ecological and temporal dimensions [20,21]. Individuals within a colony have stringent dietary requirements. Immature bees depend on a diet of bee bread, a processed food derived from pollen containing key amino acids for growth and development, while nectar is the primary energy source, to be consumed or stored as honey [22–24]. Disruption of this dietary balance can be critically detrimental to overall colony health [25–27], and affects a host of physiological and behavioral honey bee traits [28–31]. Specifically, protein deprivation increases individual mortality and disease [30], while negatively impacting learning and short-term memory [31,32]. These effects extend to sensory capabilities and affect select downstream behaviors and social cohesion [25,27,31,33,34].

Key biomarkers are established for both colony defense and nutrient deprivation. Nutritional markers *Vg, Ilp1,* and *IRS* have long been associated with nutritional deprivation and seasonal change, which has inspired use as general measures of nutritive health [35–39]. Rittschoff and Robinson (2013) [6] identified and examined four genes (*Cyp6g1/2*, *drat*, *inos*, and *GB53860)* associated with defensiveness and had differential expression when bees were exposed to acute stressors such as alarm pheromone, electric shock, and/or physical disturbance of the colony. There are some contextual nuances with these genes, as with the nutritional markers, because they can vary in honey bee caste (forager vs soldier bees) and test conditions [6,12,40]. A different study showed association of *Cyp6g1/2* and alarm pheromones [41], although aggression assay types were slightly different in this study (did not include synthetic alarm pheromone exposure) [6].

To test how environmental stressors mediate the intensity of colony defense, we examined the behavior of colonies under a distinct resource limitation. Specifically, we experimentally induced a pollen dearth for a subset of colonies kept in a common garden design. Over a five week period, we monitored changes in colony defense using an established methodology [10,14]. To better understand our colony behavior results, we also conducted a survey of physiological parameters and individual-level variation within measures of gene expression for key markers previously associated with colony defense (*Cyp6g1/2, drat, inos,* and *GB53860*) [6,41]*,* and nutrient deprivation (*Vg, Ilp1,* and *IRS*) [35–39].

## Materials and methods

### Experimental design

Sixteen Pol-line colonies were established at one apiary location near St. Gabriel, LA in the spring of 2022. Queens were reared at the USDA-ARS Honey Bee Breeding, Genetics, and Physiology Research Unit in Baton Rouge, LA. All queens were reared at the same time from the same group of breeder queens and open mated in an isolated location following well established practices [42–43]. Use of these bees reduced genetic variation, allowing closer examination of the role of environmental impacts on behavior and physiology. In August of 2022, colony population, brood, and nutritional resources were standardized to 8 deep frames of adult bees in: one brood box (containing three frames of open brood, three frames of closed brood, one empty frame, and one honey frame) and one super box (containing three empty frames empty and three frames of honey). Each colony was equipped with a Sundance Pollen Trap (Betterbee, Greenwich, NY, U.S.A).

**Fig 1:**
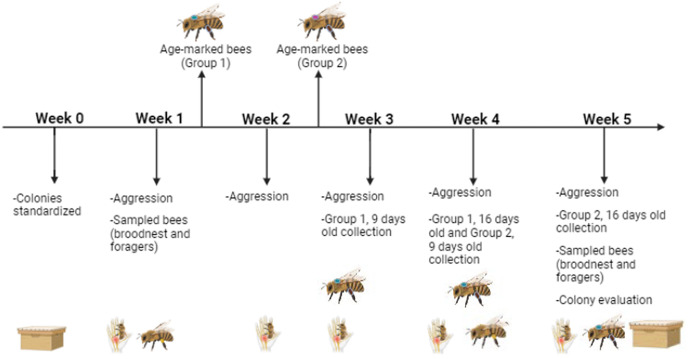
Experimental design and timeline. Age-marked bees were painted from newly emerging bees between Weeks 1 and 2, then collected at 9 or 16 days old during the aggression assays (above line). Assays and samples collected per week are described below the weekly headings. Pictograms: Colony box and lid = resource (honey, pollen, brood, population) assessment; bee stinging hand = aggression assay; Bee with pollen on corbicula = sampled bees (broodnest and forager); bee with paint mark = bees collected in Groups 1 or 2 (if below the timeline) or that emerging bees were paint marked (above the timeline), referred to as “Age-marked bees”. Image created in BioRender.

As determined by random treatment assignment, half of the colonies in the apiary were pollen deprived by means of turning the pollen traps “on” (pollen deprived colonies called “PD”) and the other half of the colonies had their pollen traps continually “off” (pollen control colonies called “PC”). Pollen was collected twice weekly from the traps throughout the six-week experiment. Colony brood bees were sampled weekly, but only analyzed for Weeks 1 and 5. To distinguish between general effects of pollen deprivation treatment or time as opposed to age of individual workers, we paint marked two cohorts of bees at their emergence (hereafter “Group 1” and “Group 2”) and reintroduced them the same day into the colonies they were reared in (Fig 1). Age-marked bees were collected at 9 and 16 days post-emergence for subsequent sampling. These bees are distinguished from each other in the figure legends and results. Subsequent molecular and physiological analyses were conducted on foragers, individual paint marked bees, and pooled samples of bees collected from the colonies (S1 Table). Bee bread was collected by manually scooping cells of bee bread from comb into 15 ml falcon tubes with a small metal spatula. All samples were frozen on dry ice in the field and put into a -80C freezer upon return to the laboratory for subsequent analyses.

### Aggression assay

To examine honey bee colony defensiveness, we applied a ratings assay as is established methodology [5,44]. Briefly, an observer examined the colony over an average of two minutes. Four specific behaviors were ranked during the observation: degree of stinging (sting), propensity of hanging from the comb (hang), magnitude of running over the comb (run), intensity of flight around observer (fly). The ranking is based on intensity of response on a 1–4 scale based on established criteria [5,44]. In this study, the assay was conducted each week at the same time by the same observer and following the same order of colonies assayed to maintain those variables as constants.

### Physiology and metabolic assays

To evaluate physiologic and metabolic markers of honey bee nutritive health, we assessed hypopharyngeal glands (HPG) and lipids of marked bees. We dissected the heads of age-marked bees for hypopharyngeal gland (HPG) analysis according to standard methodology [45]. Lipid analysis was performed on broodnest bee abdomens according established methods [46], where abdomens go through a dehydration and homogenization process to dissolve carbohydrates, and then the resulting material was made into a homogenate that reached a final solution of 2% Na_2_SO_4_ [47–48] which was then treated with chloroform-methanol solution to solubilize lipids for removal (see Supplementary Methods).

### Molecular sample processing

To evaluate general biomarkers often associated with nutritionally regulated physiological states or aggression, real-time PCR was conducted in triplicate using well established protocols [42]. Pools of 25 bees collected from the broodnest of each colony were used to assess colony level physiological markers, while individual bee heads were processed for aggression related, *Vg, Ilp1,* and *IRS* gene expression (refer to Supplemental Methods, S1 and S2 Tables). Relative gene expression was calculated using the ΔCt method following log transformation to approximate normality. Analyses of gene expression data were performed in R using the lmer() function of the lme4 package [49] and again tested the interaction of treatment and date or group with colony as the individual examined.

### Pollen analyses

Pollen and bee bread were analyzed for identification and total protein content. Pollen traps were briefly turned on for six hours for all colonies at the conclusion of the study to collect and characterize the foraged resources. Bee bread was collected via manually scooping it from cells within the colony. The pollen pellets and bee bread samples were thoroughly mixed and 0.25g of pollen from each sample was homogenized with 95% glacial acetic acid and processed with standard acetolysis procedures [50,51]. The first 200 pollen grains encountered were counted and identified to the lowest taxonomic level possible [52]. Approximately 100 mg of pollen was weighed and sent out to the Mississippi State University Soil Testing Lab for Nitrogen analysis. The samples were analyzed with a Dumas combustion assay on the Elementar vario MAX cube organic elemental analyzer and the percent N was multiplied by a conversion factor of 6.25 to estimate the total protein in the samples, as is standard practice [53].

### Statistical analyses

Past studies have used the combination of the four defensive assay ranks (of hanging, stinging, flying, and running) [5,10,44], per colony and later via multi-dimensional scaling (MDS) [11] to estimate overall defense per colony. To score utilizing this assay, the four defensive behaviors are all scored by the observer 1–4. However, if one defensive behavior scored higher than the minimum score, this method does not indicate which of the four behaviors was scored higher than the others or to what extent it differs. The MDS approach used in other studies [11] partially overcomes this limitation, however it assumes a-prior constraints in the number of dimensions. In this context SVD is advantageous because it accounts for the total sum of the four behavioral ranks while retaining nuanced differences generally lost if traditional methods like total rank summation are used behaviors (e.g., two colonies that score “6” would be treated as the same with a total rank system, but with the SVD the behaviors which scored higher is retained). The principal eigenvector in our SVD approach is therefore related to total rank sum, while still a more accurate proxy for general colony defense (S3 Fig).

**Fig 2:**
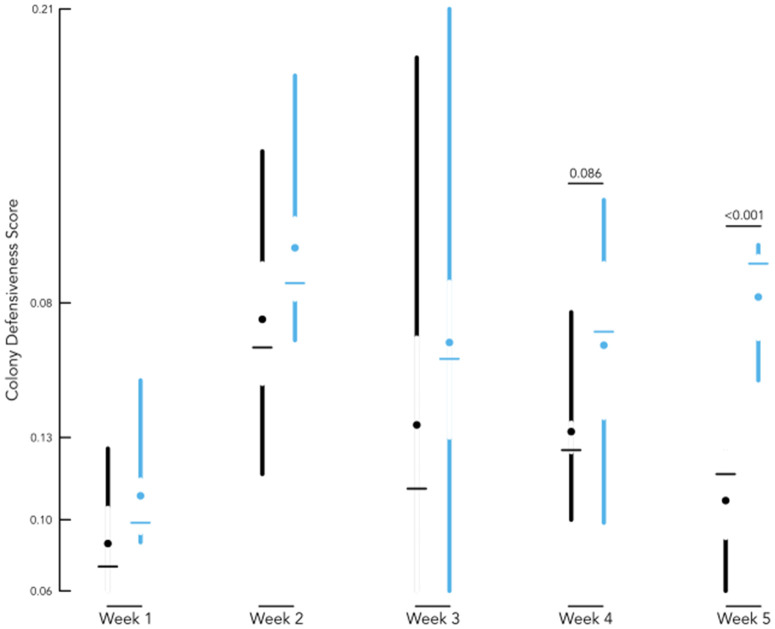
Pollen-deprived colonies had higher defensive assay scores than control colonies. The figure illustrates the distribution of factors scores across our sampling timeline and between control (black) and pollen deprived (light blue) colonies. Across all observations (N = 75) x-axis denotes discrete time points, and y-axis provides the defensive score. The y-axis extends from lowest to highest score obtained in the data set and tick marks are made at each quartile. For each group, segments extend from minima to maxima with gaps bounding the interquartile range of the data, open circles identifying the mean, and horizontal bars identifying the median. Trending and significant timepoints are indicated with the p-value above the week. Pollen deprived colonies showed a trend of more aggressive behavior (p = 0.086) than control colonies in Week 4. Pollen deprived colonies were significantly more aggressive than control colonies (p < 0.001) in Week 5.

Using the SVD eigenvector values as colony defensiveness scores, we applied them as our response variable to examine the effect of Treatment over time using a repeated measures analysis (Fig 2). Our model was fitted using the lmer() function [54] in R and tested the interaction of Treatment and Date of observation, using colony ID as the individual examined. As a post-hoc analysis of least square means, emmeans() function in R identified the degree of divergence between groups at each time point.

Estimates of colony hypopharyngeal gland (HPG) size were derived from the average of acini size across 10 bees from each colony and date set. Two groups of bees were separately examined in our analyses, broodnest bees and age-marked bees. For broodnest bees, data was examined using a linear model approach with the lm() function in R [49] with Treatment (Control, Pollen-deprived), time, and colony as fixed effect factors. For age-marked bees, we applied a similar model, but one that only considered Treatment, group cohort (Group 1 bees had their open brood stage before the experiment started and then emerged into pollen-deprived or non-deprived colony environments while Group 2 bees were reared in the experimental environment they emerged into), age, and colony as fixed effects. When appropriate we applied post-hoc analyses to determine which element in the factors considered were driving the detected differences.

We examined metabolic response through analysis of total weight, glycogen weight, and lipid weight across both broodnest and age-marked bee groups. For all contrasts we applied a linear model approach with a model structure like that described above for the HPG analyses. One exception was that for analysis of glycogen and lipid weights, we had to account for the correlation of these values and total weight. To achieve this, we added total weight as a fixed effect factor in those analyses only.

Broodnest bee analyses were linear model approaches with the lm() function in R [49] with PD and PC treatment, time, and colony as fixed effect factors. For age-marked bees, we applied a similar model, but one that only considered PD or PC treatment, group (Group 1 vs. Group 2, where Group 1 PD individuals had exposure to pollen as larvae and Group 2 PD individuals did not), age (if tested), and colony as fixed effects. When appropriate we applied post-hoc analyses to determine which element in the factors considered were driving the detected differences in significant results. At the conclusion of the study, we surveyed colony population size and resources (S2 Fig) and then examined how these related to behavioral response. Contrasts were done via two-factor ANOVA using the aov() function in R [49] with defense factor score for our final time point as the response variable and each of the four variables we examined: relative number of bees, brood, pollen, and honey area as dependent variables. Our model used treatment as the second blocking factor.

## Results

We utilized a standard experimental design, where colonies bred and established at the same time from the Pol-line population [42,43] were kept in a single location and randomly assigned as pollen-deprived (PD) or not (PC) after being fitted with pollen traps. All colonies were monitored for queenrightness and general health over the course of five weeks in the late summer with weekly sampling events and behavioral assays to quantify colony defensive response (Fig 1) [5,14]. Physiological metrics were examined in bees collected either from the broodnest within a colony, foragers as they returned, or age-marked bees. Measurements included quantification and comparison of aggression and nutritive markers, respectively *Cyp6g1/2, drat, inos*, and *GB53860* expression in addition to HPG size, body weight, lipid and glycogen levels, and *Vg, Ilp1,* and *IRS* expression (see S1 Table). Age-marked subsets bees in six colonies of each treatment group were used to contrast if aggression or nutritive markers varied based on rearing environment (where Group 1 were open brood before the experiment began and Group 2 were open brood in the experimental treatment groups) (Fig 1; S1, S2 Tables). This allowed us to examine gene expressions in bees whose precise age was known, as well as in bees based on task allocation.

Available pollen in the environment largely consisted of Asteraceae (S1 Fig) and collection by plant species, lipid, or protein content did not vary between colonies that were or were not pollen deprived at the end of the experiment. Colonies significantly differed as of the fifth week of the experiment in frames of pollen (t = 4.68, p = < 0.000), brood (t = 2.79, p = 0.0153), and adult populations present in the colonies (t = 3.61, p = 0.0032), but not in frames of honey, indicating that the pollen trap treatment sufficiently created a significant pollen dearth for the pollen deprived Treatment colonies (S2 Fig).

### Nutritional restriction effects on colony defense

Colony defense was monitored on a weekly basis for each colony over the course of the five week experimental period. Intensity of defensiveness was measured following established methods [5,14], whereby rankings were assigned individually to four bee behaviors (e.g., stinging, hanging, flying, and running). In lieu of a total rank we applied a single value decomposition (SVD) of the sample x rank matrix. This allowed us to extract the first eigenvector as our colony defensiveness score. The SVD significantly correlated with the sum total rank (R2 = 0.98, t-value = −67.495, p = 2.2E-16; S3 Fig), but allowed us to retain nuances in colonies that had equal total scores but distinct distributions across the.

We next conducted an analysis of the colony defensiveness score, which was significantly affected by both pollen deprivation status (Linear Mixed Effect F1 = 10.03, p = 7.42E-3) and time throughout the study (Linear Mixed Effect F4 = 9.38, p = 7.08E-6) without an interaction between these variables (Fig 2). Post-hoc analysis identified that PD and PC colonies at each specific time point showed overall differences between defensive scores. These were largely driven by the last colony check at Week 5 (Fig 2), where PD colonies had much higher aggression scores than PC colonies. Focusing on week five, we saw a significant divergence between PD and PC colonies (LM coefficient estimate = 0.05429, t-value = 6.845, p = 1.18E-5), which was not present in the initial three weeks and was only a marginal trend at Week 4 (Fig 2).

**Fig 3:**
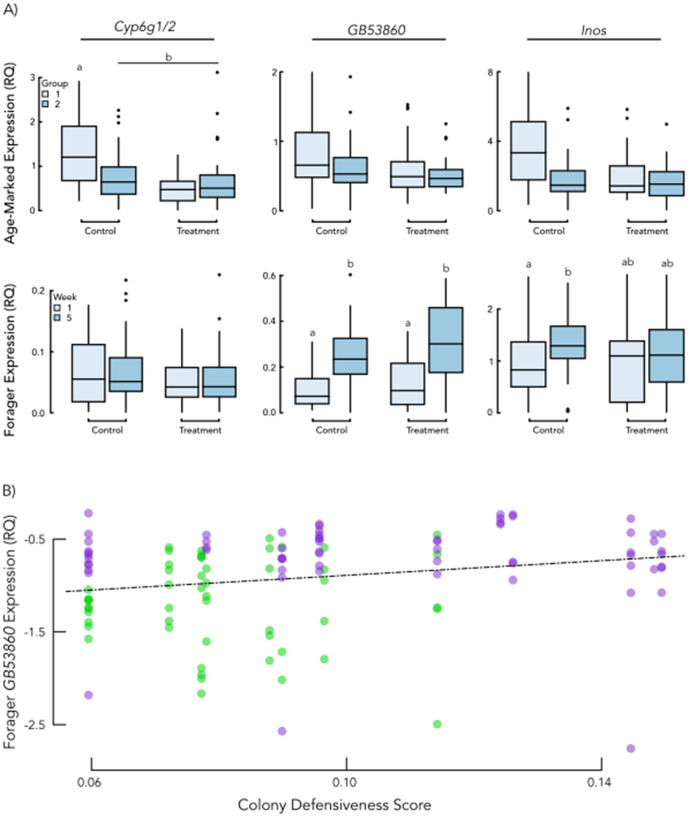
Behavior-associated gene expression. The figure illustrates associations between colony defensive behavior and target genes previously associated with the trait. Within panel A, light blue denotes Group 1 individuals and darker blue denotes Group 2 individuals. “Control” refers to colonies that had continual access to all pollen foragers returned with while “Treatment” refers to colonies that had activated pollen traps and were pollen deprived. In (A) measures of gene expression are highlighted for all targets and indicated effects of treatment, time and rearing environment for age-marked bees (top) and foragers (bottom). Significant differences between groups (p < 0.05) are indicated with different letters above the box plots. Cyp6g1/2, GB53860, and inos are all genes historically associated with significant changes in expression in individual bees with high aggression. Expression of Cyp6g1/2 in heads of 16d old age-marked bees was significantly reduced by Treatment in Group 1 bees, while expression in Group 2 bees was low overall (N = 136). Profiles of GB53860 (N = 133) and inos (N = 126) showed expression in age-marked bees was not significantly influenced by Group or pollen deprivation treatment. Among foragers, Cyp6g1/2 expression did not differ due to time or treatment (N = 133), though GB53860 expression increased generally from week 1 to week 5 (N = 146). Expression of inos in foragers increased across time in bees from pollen control colonies, while those from pollen deprivation colonies maintained similar expression across weeks (N = 149). Illustrated in panel (B) is the relationship between GB53860 and Colony Defensiveness Score in foragers. In the panel figure the x-axis corresponds to our Colony Defensiveness Score and they y-axis to the relative expression level for GB53860, with each point indicating an individual bee. The dashed line highlights the observed correlation while the colors delineate time point with green for Week 1 samples and purple for Week 5 samples. Across our panel of gene targets, GB53860 was the only one whose expression level significantly correlated with our measure of defense.

We also investigated key biomarkers of colony defense through gene expression of a panel of targets (*Cyp6g1/2, drat, inos*, and *GB53860*) previously associated with aggression and defensive behaviors [6]. Specifically, we analyzed individual heads of 16 day old age-marked bees and foragers. Within age-marked bees, we assessed two distinct subgroups: individuals who were pupae when pollen traps were applied and thus only experienced pollen deprivation as adults after emergence (Group 1) and individuals that were exposed to pollen deprivation at both the larval and adult stages (Group 2) (Fig 1). In age-marked bees, we saw significant interaction between Group and PD or PC treatment variables for *Cyp6g1/2* expression (F1 = 9.45, p = 0.02), with Group 1 individuals from PD treatment colonies having significantly greater expression levels than all other Group x Treatment combinations (Fig 3A). In contrast for *drat, inos*, and *GB53860*, expression did not differ significantly based on pollen deprivation treatment, Group, or the interaction (p > 0.05) among age-marked bees (Fig 3A). Among the foragers collected, there were no significant differences in *Cyp6g1/2* or *drat* expression (Fig 3A) based on pollen deprived treatment or control colonies. However, *GB53860* expression significantly differed over time (F1 = 14.50, p = 0.0002) with increased expression at week 5 (Fig 3A). Furthermore, for *inos* expression there was a significant interaction between pollen deprivation treatment and week (F1 = 4.23, p = 0.042). Over time, *inos* expression increased in the PC colonies, whereas PD colonies maintained similar *inos* expression (Fig 3A). A correlative analysis was also conducted with colony defensiveness score, and against this variable, *GB53860* showed a significant positive correlation (Generalized Linear Model, F1 = 6.573, p = 0.0117; Fig 3B) where individuals from highly defensive colonies also had high expression.

### Nutritional restriction effects on physiology

**Fig 4:**
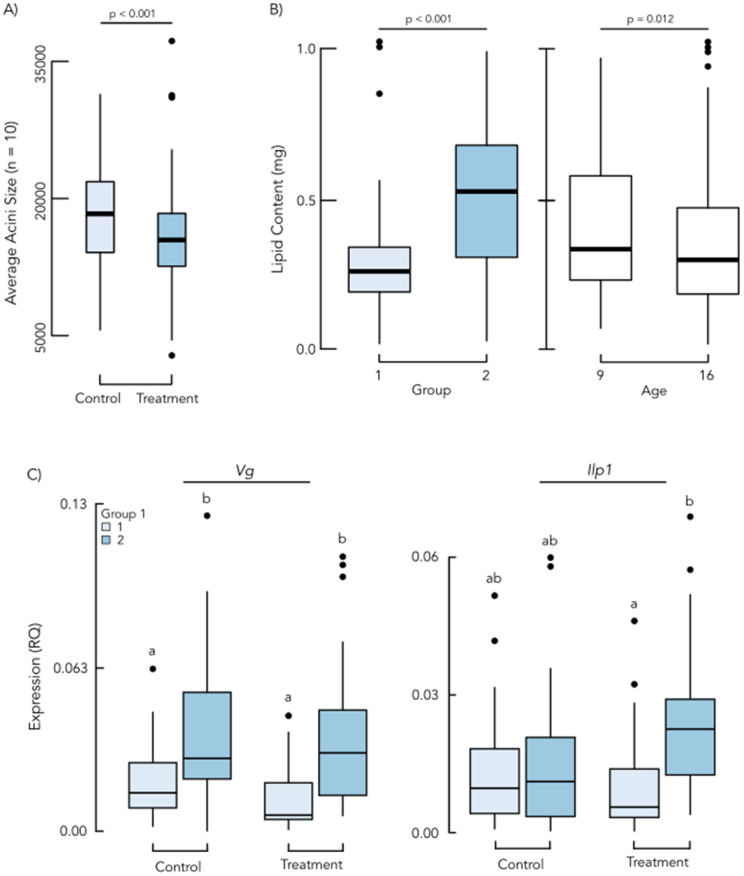
Physiological and metabolic assessment of groups. The figure illustrates an analysis of multiple physiological and metabolic parameters including gene expression for honey bees sampled in this study. Panel (A) highlights differences observed in Hypopharyngeal gland (HPG) size of brood nest bees with overall lower size measured in the Treatment (pollen deprived) group in Week 5. In panel (B) differences in lipid content of age-marked bees is demonstrated, with two independent effects observed between rearing groups and age (days old). Between groups, there was significantly greater lipid content in bees that experienced pollen deprivation throughout their entire developmental cycle (Group 2; dark blue) compared to Group 1 (light blue). Across ages, we saw an overall decrease of lipid content independent of group membership. Panel (C) illustrates results from gene expression analyses in age-marked bees where Vg expression showed a Group effect independent of PD or PC treatment, while Ilp1 showed significant differences only in the pollen deprived treatment Group 2. In panel C, light blue denotes Group 1 and dark blue denotes Group 2.

Investigation of changes in physiological parameters included physical measures, metabolic assessments, and gene expression of key targets (*Vg, Ilp1, IRS*). Specifically for physical measures, we examined changes in average HPG size and total weight across broodnest bees within PD and PC colonies over the experimental period. We found that HPG size independently correlated with PD treatment (F1 = 11.85, p = 7.03E-3) and time (F4 = 2.82, p = 0.026; Fig 4A) with PC bees generally achieving larger HPG sizes than those in the PD colonies. A post-hoc analysis of the effects of time showed this was due to an overall decrease in HPG size for bees collected on Week 4 (t199 = −3.031, p = 0.010). A similar assessment of acini in the HPG of age-marked bees, showed significant differences across Groups (F1 = 21.60, p = 8.56E-6; Fig 4A) with marginal trends across PD and PC treatments (F1 = 3.17, p = 0.077) in addition to overall age of bees (F1 = 3.48, p = 0.065). In contrast, total bee weight showed no effect of PD or PC treatment (F1 = 1.45, p = 0.229) or time (F4 = 1.56, p = 0.184) in broodnest or age-marked bees (F1 = 3.48, p = 0.064).

Given the close ties between HPG size and nutritional status, we followed our analysis with an assessment of glycogen and lipid contents of bees based on PC or PD status, with mixed results. Estimates of glycogen content within broodnest bees showed a significant effect of time (F4 = 17.11, p = 1.74E-12), where the biggest difference was a transient increase in levels of glycogen in broodnest bees collected at Week 2 (t263 = 5.88, p = 5.07E-8). Concordant analyses of lipid levels in broodnest bees identified a significant interaction effect between PD or PC treatment and time variables (F4 = 3.80, p = 5.21E-3), driven by higher overall lipid levels in the control colony bees for Weeks 3 and 4. Within age-marked bees, PD and PC treatment effects were detected for both estimates with glycogen levels significantly lower in Group 2 (F1 = 42.28, p = 1.53E-9) while lipid content was inverted and Group 2 showed the highest levels in this measure (F1 = 39.0, p = 6.28E-9, Fig 4B).

Further examination of key physiological biomarkers was applied through analysis of gene expression for a panel including genes: *Vg, Ilp1* and *IRS*. These genes are established indicators of physiological health and seasonality, and therefore very sensitive to nutritional status [32–36]. In age-marked bees, *Vg* was influenced by group with Group 2 having increased expression (χ2 = 29.83, p < 0.001; Fig 4C), with no effect of PD or PC treatment or interaction with Group. *Ilp1* was affected by the interaction between PD or PC treatment and Group (χ2 = 9.93, p = 0.002). *Ilp1* was higher in Group 2 (larvae development took place in pollen deprived or pollen control rearing environments) than Group 1 (only the adult stage of the individuals was exposed to pollen deprived or pollen control environments) in the PD treatment colonies (emm = −0.017, p < 0.001; Fig 4C). *IRS* expression in age-marked bees exhibited no effects of Group or PD or PC treatment (p > 0.1). Analysis of a pool of 25 broodnest bees per colony indicated significant effects of time, but not PD or PC treatment or the treatment interaction with time, for *Vg, Ilp1*, and *IRS* with increased gene expression levels over the experimental period (*Vg*: χ2 = 46.44, p < 0.001; *Ilp1*:χ2 = 12.78, p < 0.001; *IRS*: χ2 = 20.45, p < 0.001; S4 Fig). It is worth noting that the experimental time period took place in the fall.

## Discussion

The principal interest in our study was to determine the effect of an environmental variable (food availability) on group defensive behavior, in addition to conducting a closer look at potential mechanisms or physiological drivers for these behaviors. For all colonies in our study, colony defensiveness scores initially increased over the experimental period. Differences between experimental groups became evident over the last two time points and culminated in colonies with significantly increased aggression (Fig 1). The initial increase in intensity for the behavior in Week 2–3 may be due to an increase in handling or tied to resource (brood, honey) guarding as colonies increased in size. However, we did see a decrease in intensity as time goes on in PC colonies. This suggests that PC colonies returned to baseline temperament while PD colonies became or maintained “hangry” status as their deprivation continued. Hanger is a portmanteau combining “hungry” and “angry,” the word describes an increase in aggression caused by hunger—a phenomena also generally observed in other species [55–57]. Changes in colony defensiveness are of particular concern to beekeepers, as increased defensiveness also increases safety risk of personnel and potentially impacts regulatory practices.

Ultimately, this study shows that both researchers and farmers must find ways to evaluate how livestock perform in changing environments in order to both form best management practices and make breeding decisions. Genetic background correlated with phenotype is the main breeding criteria for commercially kept livestock species, from plants to insects to mammals [58–59]. As extended periods of nutritional dearth are now commonplace across the world, it becomes more important to re-evaluate behavioral traits like temperament in the context of a changing environment. In this study the simulated pollen dearth caused a significant decrease in frames of adult population, brood, and pollen at the end of our experiment (S2 Fig), which is expected. Dramatic behavioral changes to the extent seen in this study were not, although we have investigated further in attempts to explain this behavioral shift.

Defensive behaviors in honey bees are a matter of high concern to both beekeepers and policy makers, to the point that some regions of North America do not allow importation of bees which may have genetic backgrounds associated with aggression, namely *Apis mellifera scutellata* and hybrids as determined by molecular assessment. This has necessitated beekeepers to take genetic background into account while breeding bees, sometimes to the exclusion of other traits. Behaviors (i.e., *Varroa* Sensitive Hygiene) are generally considered more ideal breeding criteria when possible because of the potential economic savings in decreasing colony mortality.

It is known that periods of larval starvation cause behavioral [27,31,60,61] and physiological [60–63] effects in the resulting adults, which is known as a priming effect. These effects sometimes carry beyond individuals and can cause transgenerational [20] or colony affects. When we examined individual bees from experimental colonies, we found that there may be evidence of a priming effect due to the larval rearing environment. This is supported by the possibility of priming effects between aggression and other variables [16] as well. Our study examined possible effects of priming by contrasting two distinct groups of age-marked bees (Fig 1), where Group 1 matured in similar environments and Group 2 in environments that were already either pollen deprived or pollen control colonies. This allowed us to survey gene expression in individuals who experienced different larval rearing environments (Group 1 had all experience pollen-rich rearing environments as larvae while Group 2 had half of the individuals experience pollen deprivation as larvae). Using a biomarker set previously associated with colony defense [6], we identified differences in only one gene, *Cyp6g1/2*, for only Group 1 bees across experimental groups, with PC bees having overall higher gene expression (Fig 3A). Both groups of age-marked bees were collected once they were at the same age in our study. This result suggest that any mediation of colony defense by *Cyp6g1/2* is contextually dependent on rearing environment, a point that may explain inconsistencies in outcomes across studies looking at this gene’s relationship to colony defense [6,40,41]. These findings illustrate the possibility of additional nuance between acute and chronic stressors utilized in these various studies.

The expression profile of our behavioral gene panel in foraging bees also showed a degree of nuance. Gene expression of *GB53860* increased over time in this study. While it did not show an association with nutritional treatment, it highly correlated with defensive scores (Fig 2, 3B). This means that the function of *GB53860* may be insensitive to nutritional stress directly, but appears to be a robust indicator of a state of aggression or defensive behavior. In contrast *drat*, which differs in previous work [6,40,41], did not show any significant differences in this study. This potentially suggests stressor-specific sensitivity (i.e., to acute colony disturbance) rather than a broad association colony defense level. Expression of *inos* was similarly complex with clear interaction effects detected between treatment and time factors, demonstrated by expression variation across time for PC foragers but no such patterns for PD foragers. This finding is consistent with previous work associating a downregulation of *inos* with increased defensive behavior [6,12]. Overall, gene expression results highlight that there are nuances across stressor types and development which need to be accounted for in further analysis of these targets.

Physiological measures similarly show a complex relationship between dearth, and metabolic response. For instance, the effects of pollen deprivation and time each had a separate effect on acini size in HPG of broodnest bees (Fig 4A). Age-marked bee groups reared under different colony nutritional environments also had different HPG sizes, which would be expected in different ages but not based on rearing Group environment. This finding seems to support the hypothesis that immature bees receive rearing cues [60–63] which prepare them physiologically to experience a similar environment as adults, or have a functional priming effect. This pattern is also present in the age-marked bee lipid and glycogen levels which also had differences between Groups (Fig 4B) and had additional differences over time and PD or PC treatment in pooled broodnest bees.

The biomarkers *Vg, Ilp1*, and *IRS* in pooled broodnest bees all showed increased expression over time that was insensitive to experimental disturbance (S4 Fig). The change over time during a region’s fall season of *Vg, Ilp1*, and *IRS* [36,64] suggests that the subtropical climate where this experiment was conducted has the similar timeline for producing winterized bees as more temperate areas [20,65]. This probable effect of seasonality likely also drove the difference in *Vg* expression in the age-marked bees across Group 1 and Group 2 (Fig. 4C), potentially overwhelming any differing signal from the experimental treatment groups and explaining why there is no observed significance based on nutritional grouping. A specific finding of interest is that in this experiment, bees showed elevated expression of *Vg* despite reduced pollen resources. This has already been observed due to seasonal brood rearing patterns [66], but our results add another layer between the defensive behaviors and the seasonal maintenance of the colony. It seems clear that there is a complex allocation of resources at behavioral, physiological, and colony levels which is highly contextual based on the environment.

Many various factors are needed to get an accurate idea of the effect of relative factor importance, interactions, and their resulting impact. For instance, we knew that pollen deprivation could lead to dramatic physiological changes in individual bees. However, increased defensive behavior as a downstream consequence of nutritional deprivation was not formerly known. Furthermore, our work highlights that gene expression variables corresponding to defensive behaviors need further refinement. Additional work could discover if changes in expression profile are due to sensitivity to stressors, ecological context, or are, like *GB53860*, stable associations with defense scores. These results also support the conclusion that there are environmental cues immature bees receive which may physiologically prime them for the environment they will encounter as adults [60–63]. It appears that changing the environment after larval maturation and before adult emergence can mediate changes in individual temperament and other physiological measures, which ultimately is most visible through increased colony defensive behavior.

## Conclusion

On a broader scale, our work shows that bees of very similar genetic background can become measurably more defensive than genetically similar colonies when they encounter a stressor event from the environment, in this case food deprivation showed an increase in defensive behaviors which could be described as “hanger.” As the Anthropocene continues, unpredictable major weather events—including droughts, hurricanes and variability in environmental resources—will intensify and livestock management practices need to adjust in order for food security to be preserved [67–68]. This may necessitate a shift in evaluation techniques relating to honey bee aggression and breeding, as this study makes it clear that holistic evaluation techniques which include behavior assays give insights beyond simply determining genetic background. It is imperative that policy makers keep a holistic evaluation mindset when forming regulations in order to meaningfully form science-based policies that meet public goals.

## Supporting information

S1 Table Overview of analyses done for each bee sample collected. A visual representation can be found in Fig. 1. Age-marked bees were collected at 9d and 16d post-emergence because of the physiological changes in HPG, lipid, glycogen, and total weight changes that are expected over time as bees age and transition to different tasks. These are general health markers that are often associated with nutrition. Vg, Ilp, and IRS, are also thought of as general health markers that coorespond to nutrition level available to the individual. Aggression biomarkers Cyp61/2, drat, inos, and GB53860 have been associated with bees that have displayed aggressive behaviors.
(PDF)

S2 Table Real-time PCR primer information and thermal protocols. Target names, primers, target type, annealing temperature, gene ID, and reference are listed.
(DOCX)

S1 Fig Protein content and pollen identification collected from all colonies over a half day period at the conclusion of the experiment. There was no difference in protein content between pollen collected by either Control and Treatment, and the dominant pollen type for all colonies was Asteraceae.
(PDF)

S2 Fig Colony population and resources at the conclusion of the experiment. Treatment colonies had significantly fewer frames of adult bees, brood, or pollen than control counterparts. There was no difference in frames of honey.
(PDF)

S3 Fig Relationship between total rank and colony defensiveness scores. The plot highlights the correlation between the SVD-derived eigenvector “Colony Defensiveness Score” and the total sum of behavioral ranks. Initial relationship was inverted with score decreasing as rank increased so a correction was applied to facilitate interpretation of values in follow-up statistical analyses.
(PDF)

S4 Fig Expression of Vg, Ilp1, and IRS in broodnest bees from weeks 1 and 5. (PDF)
