# Supplementary material for: Hangry bees: Pollen dearth impacts honey bee (Apis mellifera) behavior and physiology
Source: PLoS One. 2026 Jan 16;21(1):e0338712. doi: 10.1371/journal.pone.0338712 (PMC12810904; doi:10.1371/journal.pone.0338712)
Supplement: S1 Table — A visual representation can be found in Fig. 1. Age-marked bees were collected at 9d and 16d post-emergence because of the physiological changes in HPG, lipid, glycogen, and total weight changes that are expected over time as bees age and transition to different tasks. These are general health markers that are often associated with nutrition. Vg, Ilp, and IRS, are also thought of as general health markers that coorespond to nutrition level available to the individual. Aggression biomarkers Cyp61/2, drat, inos, and GB53860 have been associated with bees that have displayed aggressive behaviors. (PDF) [file pone.0338712.s001.pdf]

Supplemental Information

Bee Type	Nutritional status biomarkers		Aggression biomarkers
	HPG, lipid, glycogen, total weight	*Vg, Ilp1, IRS*	*Cyp6g1/2, drat, inos, GB53860*
**Broodnest**	Week 1, Week 5	Week 1, Week 5 (pooled samples)	
**Foragers**	Week 1, Week 5		Week 1, Week 5
**Age-marked** ***9d***	Group 1, Group 2	Group 1, Group 2 (heads only)	Group 1, Group 2 (heads only)
16d	Group 1, Group 2		

Supplementary Table S1. Overview of analyses done for each bee sample collected. A visual representation can be found in Fig. 1. Age-marked bees were collected at 9d and 16d post-emergence because of the physiological changes in HPG, lipid, glycogen, and total weight changes that are expected over time as bees age and transition to different tasks. These are general health markers that are often associated with nutrition. *Vg, Ilp,* and *IRS*, are also thought of as general health markers that coorespond to nutrition level available to the individual. Aggression biomarkers *Cyp61/2, drat, inos,* and *GB53860* have been associated with bees that have displayed aggressive behaviors.
