# Supplementary material for: Hangry bees: Pollen dearth impacts honey bee (Apis mellifera) behavior and physiology
Source: PLoS One. 2026 Jan 16;21(1):e0338712. doi: 10.1371/journal.pone.0338712 (PMC12810904; doi:10.1371/journal.pone.0338712)
Supplement: S2 Table — Target names, primers, target type, annealing temperature, gene ID, and reference are listed. (DOCX) [file pone.0338712.s002.docx]

**Supplementary Table S2. Real-time PCR primer information and thermal protocols.** Target names, primers, target type, annealing temperature, gene ID, and reference are listed.

| **Target** | **Fwd** | **Rev** | **Target Type** | **Annealing Temp (**°C**)*** | **Gene ID** | **Reference** |
| --- | --- | --- | --- | --- | --- | --- |
| Ndufa38 | GCA CGA TTC ACC AAG ACC AA | GGT TGG AGC TAC AGG CTC AGG | Reference | # 59 | XM_392983 | Cameron et al. 2013 |
| Pros54 | TCG AAC CAA GAT GGT ACT GGA A | TTG TTG TGC TTG CAG TCG TG | Reference | ## 55 | XM_393112 | Cameron et al. 2013 |
|  |  |  |  |  |  |  |
| Ilp1 | TAG GAG CGC AAC TCC TCT GT | TTCCAGAAATGGAGATGGATG | Physiology | ## 50 | XM_026442143 | Wheeler et al. 2006 |
| IRS | AAC CTT GTC GCT GCC CTT AG | GTTGGATGACTTTCGCTGGT | Physiology | ## 55 | XM_006565041 | Wheeler et al. 2006 |
| Vg | GTT GGA GAG CAA CAT GCA GA | TCGATCCATTCCTTGATGGT | Physiology | ## 52.5 | AJ517411 | Salmela et al. 2016 |
|  |  |  |  |  |  |  |
| Cyp6g1/2 | TTC GCA AAT ATC CAC CTC TAG GA | TCTATCGTCACATCAGAATTGGGT | Aggression | ### 60 | GB_52023 | Harrision, et al. 2019 |
| drat | CAC GAC ATC ACG CAG CCT T | CCTTGATCACGAACACCACG | Aggression | ### 60 | GB_55016 | Harrision, et al. 2019 |
| GB_53860 | GAACGCAAAGAGCAACCGA | CCATCCGCAGGGTGTATCATA | Aggression | ### 60 | GB_53860 | Harrision, et al. 2019 |
| inos | CCATTAGTACCACGAGGAACGC | TTTCAATTGCAGCTCGTTGTCT | Aggression | ### 60 | GB_51125 | Harrision, et al. 2019 |

*All PCR reactions were conducted on a Bio-rad CFX Connect using SsoAdvanced Universal SYBR Green Supermix following the manufacter’s recommended reaction mix for 10µl total volume. All primers were included in the mix at a 1:1 ratio.

Reactions were done using the following thermal protocols, varying at the annealing temperature:

# 95°C for 5 minutes, 40 cycles of [95°C for 5 seconds, (Annealing temp) for 10 seconds], melting curve analysis

## 95°C for 5 minutes, 40 cycles of [95°C for 5 seconds, (Annealing temp) for 10 seconds, 72°C for 10 seconds], melting curve analysis

### 95°C for 5 minutes, 40 cycles of [95°C for 5 seconds, (Annealing temp) for 45 seconds], melting curve analysis

**References**

Cameron, R.C., Duncan, E.J. & Dearden, P.K. (2013) Stable reference genes for the measurement of transcript abundance during larval caste development in the honeybee. *Apidologie* **44**, 357–366

Harrison, J. W., Palmer, J. H. & Rittschof, C. C. (2019). Altering social cue perception impacts honey bee aggression with minimal impacts on aggression-related brain gene expression. Scientific Reports, 9(1), 14642.

Salmela, H., Stark, T., Stucki, D., Fuchs, S.,... & Sundström, L. (2016). Ancient duplications have led to functional divergence of vitellogenin-like genes potentially involved in inflammation and oxidative stress in honey bees. *Genome Biology and Evolution*, *8*(3), 495-506.

Wheeler, D. E., Buck, N. & Evans, J. D. (2006). Expression of insulin pathway genes during the period of caste determination in the honey bee, Apis mellifera. *Insect molecular biology*, *15*(5), 597-602.
