# Supplementary material for: Hangry bees: Pollen dearth impacts honey bee (Apis mellifera) behavior and physiology
Source: PLoS One. 2026 Jan 16;21(1):e0338712. doi: 10.1371/journal.pone.0338712 (PMC12810904; doi:10.1371/journal.pone.0338712)
Supplement: S1 Fig — There was no difference in protein content between pollen collected by either Control and Treatment, and the dominant pollen type for all colonies was Asteraceae. (PDF) [file pone.0338712.s003.pdf]

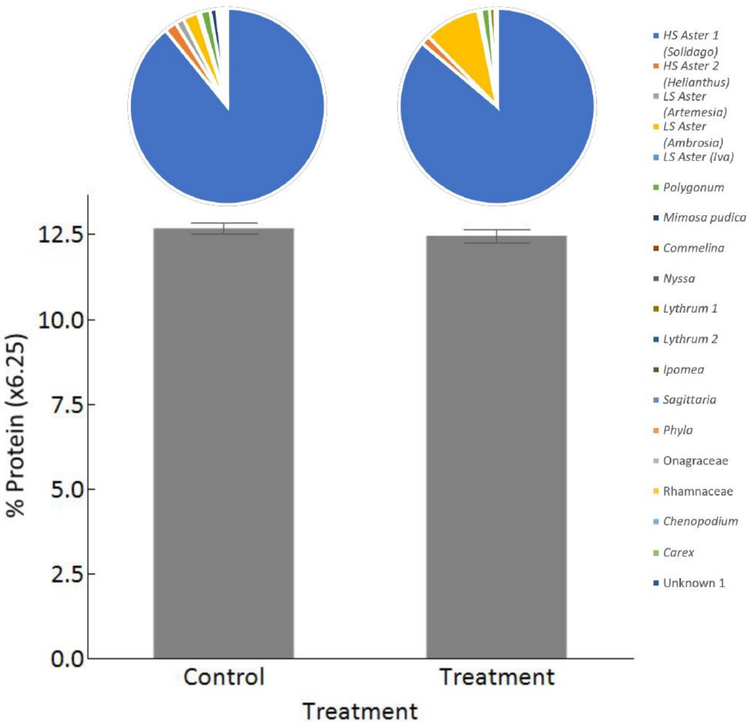

Supplemental Figure 1: Protein content and pollen identification collected from all colonies over a half day period at the conclusion of the experiment. There was no difference in protein content between pollen collected by either Control and Treatment, and the dominant pollen type for all colonies was Asteraceae.
