# Supplementary material for: Hangry bees: Pollen dearth impacts honey bee (Apis mellifera) behavior and physiology
Source: PLoS One. 2026 Jan 16;21(1):e0338712. doi: 10.1371/journal.pone.0338712 (PMC12810904; doi:10.1371/journal.pone.0338712)
Supplement: S2 Fig — Treatment colonies had significantly fewer frames of adult bees, brood, or pollen than control counterparts. There was no difference in frames of honey. (PDF) [file pone.0338712.s004.pdf]

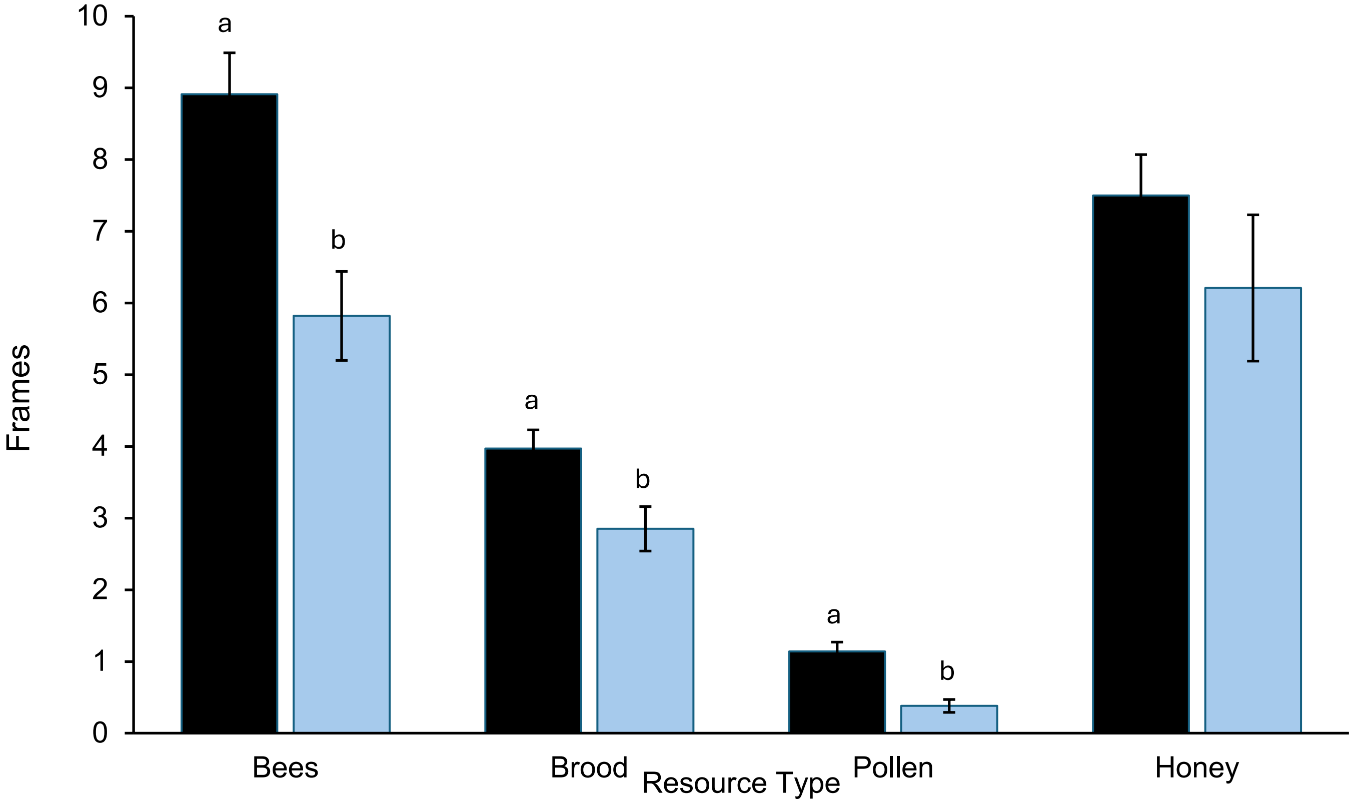

Supplemental Figure 2: Colony population and resources at the conclusion of the experiment. Treatment colonies had significantly fewer frames of adult bees, brood, or pollen than control counterparts. There was no difference in frames of honey.
