# Supplementary material for: Hangry bees: Pollen dearth impacts honey bee (Apis mellifera) behavior and physiology
Source: PLoS One. 2026 Jan 16;21(1):e0338712. doi: 10.1371/journal.pone.0338712 (PMC12810904; doi:10.1371/journal.pone.0338712)
Supplement: S3 Fig — The plot highlights the correlation between the SVD-derived eigenvector “Colony Defensiveness Score” and the total sum of behavioral ranks. Initial relationship was inverted with score decreasing as rank increased so a correction was applied to facilitate interpretation of values in follow-up statistical analyses. (PDF) [file pone.0338712.s005.pdf]

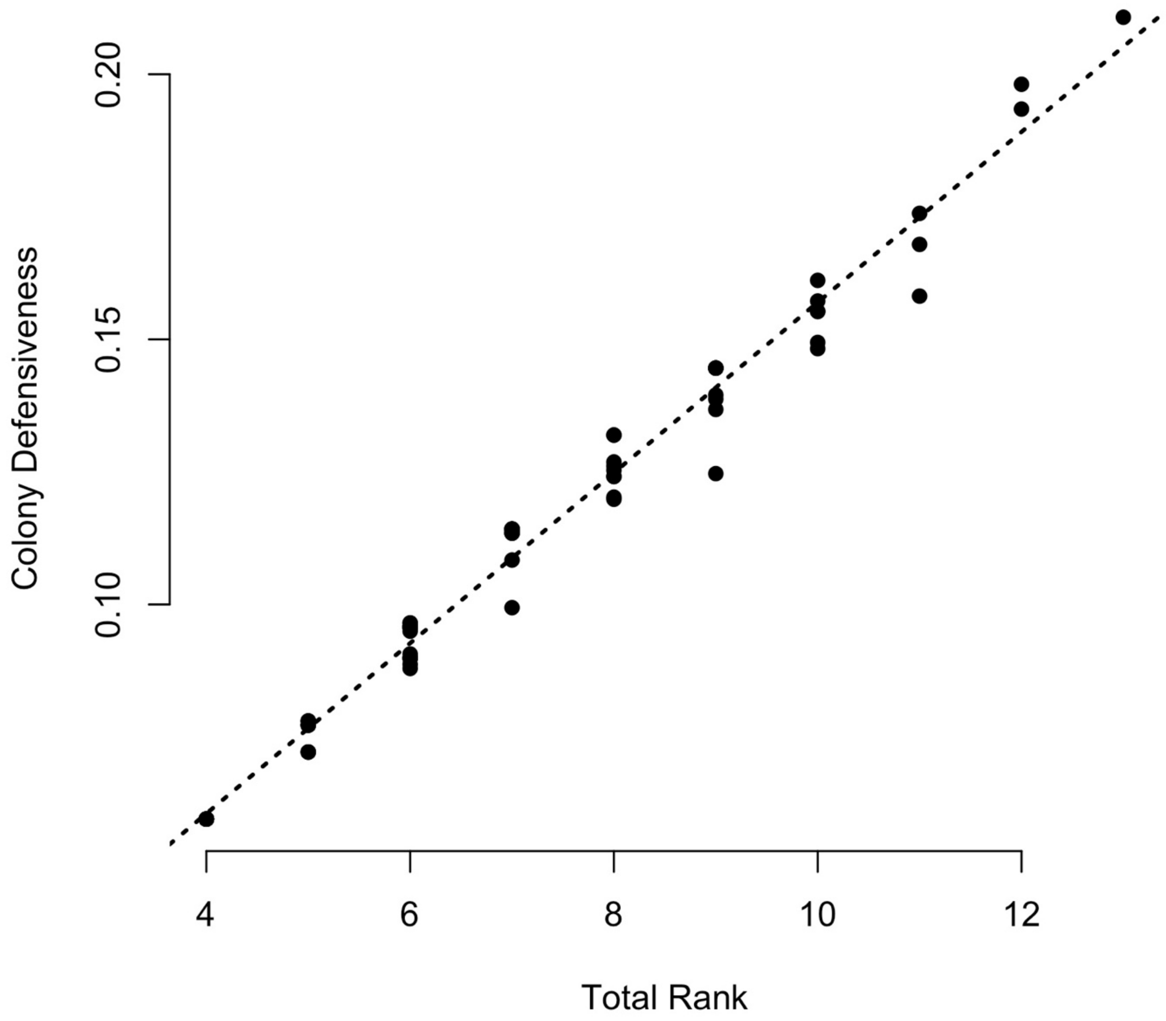

Supplemental Figure 3: Relationship between total rank and colony defensiveness scores**.** The plot highlights the correlation between the SVD-derived eigenvector "Colony Defensiveness Score" and the total sum of behavioral ranks. Initial relationship was inverted with score decreasing as rank increased so a correction was applied to facilitate interpretation of values in follow-up statistical analyses.
