# Supplementary figures and images for: Hangry bees: Pollen dearth impacts honey bee (Apis mellifera) behavior and physiology
Source: PLoS One. 2026 Jan 16;21(1):e0338712. doi: 10.1371/journal.pone.0338712 (PMC12810904; doi:10.1371/journal.pone.0338712)

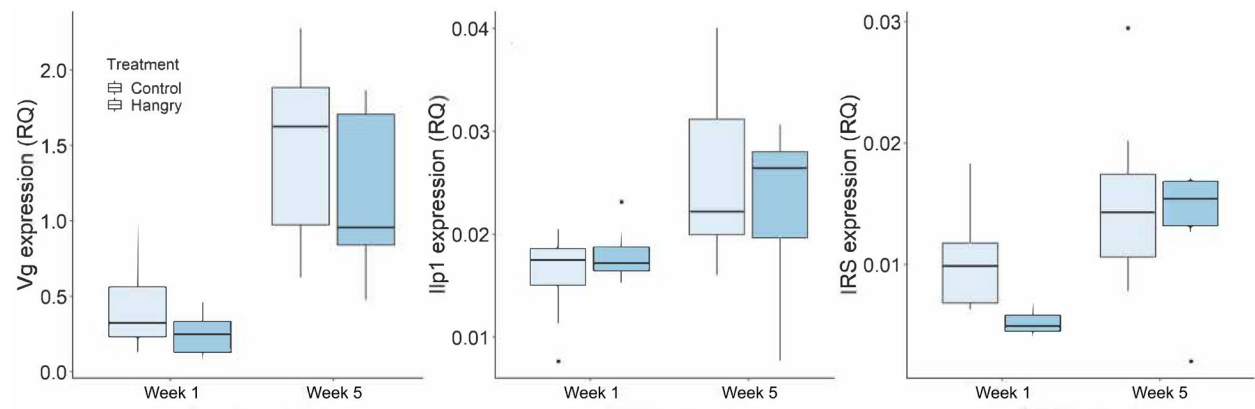

Supplemental Figure 4: Expression of Vg, Ilp1, and IRS in broodnest bees from weeks 1 and 5.

Supplement: S4 Fig — (PDF) [file pone.0338712.s006.pdf]
